# Bouveret Syndrome: A rare form of gallstone ileus a case report^☆^

**DOI:** 10.1016/j.ijscr.2024.109438

**Published:** 2024-02-28

**Authors:** S. Atri, R. Elaifia, A. Sebai, M. Hammami, A. Haddad, J.M. Kacem

**Affiliations:** Department of Surgery A La Rabta Hospital, Tunis, Tunisia; Faculty of medicine of Tunis, Tunis El Manar University, Tunis, Tunisia

**Keywords:** Cholecystoduodenal fistula, Gastric outlet obstruction, Case report, Laparotomy, Endoscopy

## Abstract

**Introduction and importance:**

Bouveret Syndrome, a rare form of gallstone ileus, involves the migration and impaction of a gallstone in the duodenum or stomach, causing gastric outlet obstruction. Early intervention and a comprehensive care plan are essential for favorable outcomes.

**Case presentation:**

This article presents a case of an 82-year-old female with a history of coronary artery disease and untreated gallstones. The patient experienced nausea, vomiting, and abdominal pain for two weeks. Diagnostic procedures revealed a cholecystoduodenal fistula with a 4 cm stone lodged at the duodenojejunal angle. For our patient the gallstone was moved to the jejunum, followed by enterotomy and a latero_lateral gastroenteroanastomosis.

**Clinical discussion:**

The rarity of Bouveret Syndrome and its nonspecific symptoms make diagnosis challenging, necessitating differentiation from other gastrointestinal disorders. Esophagogastroduodenoscopy (EGD) and imaging, such as computed tomography (CT), play crucial roles in diagnosis. In this case, the EGD did not show gallstones up to the second part of the duodenum.

Management involves a multidisciplinary approach, with supportive care for stabilization and the primary goal of removing the impacted stone. Treatment options include endoscopic, surgical, or lithotripsy techniques. Bouveret Syndrome poses challenges due to its rarity, leading to delayed diagnosis. Prognosis varies based on factors such as stone size, location, and overall patient condition.

**Conclusion:**

Through this case we emphasizes the importance of awareness, timely diagnosis, and appropriate management, with EGD and CT scan playing key roles in diagnosis. Surgical intervention remains a viable treatment option when endoscopic approaches are unavailable. The article highlights the controversial nature of fistula repair in Bouveret Syndrome.

## Highlights

• Bouveret Syndrome, a rare form of gallstone ileus causing gastric outlet obstruction.
• Esophagogastroduodenoscopy (EGD) and especially computed tomography (CT) showing Rigler triad, play crucial roles in diagnosis
• Bouveret Syndrome poses challenges due to its rarity, leading to delayed diagnosis.
• Treatment options include endoscopic, surgical, or lithotripsy techniques.
• Prognosis varies based on factors such as stone size, location, and overall patient condition.

## 1 Introduction

Bouveret Syndrome, an infrequent variant of gallstone ileus. First identified by Leon Bouveret in 1896 [1], this syndrome is characterized by the migration and impaction of a gallstone within the duodenum or stomach, leading to a mechanical obstruction of the gastrointestinal tract. In this article, we explore the various aspects of Bouveret Syndrome, from its clinical presentation to diagnostic methods and treatment strategies.

This case report has been reported in line with the SCARE Criteria [2].

## 2 Case report

**Fig. 1:**
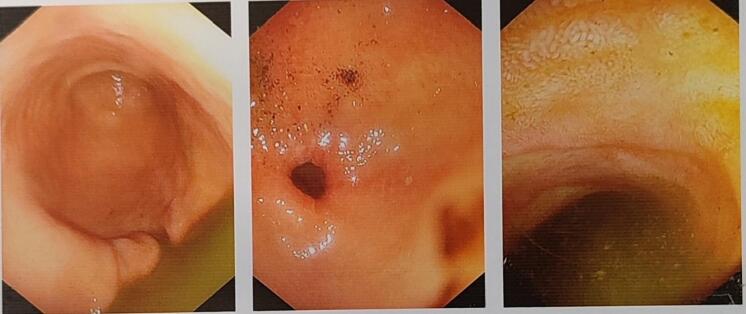
EGD to the second part of the duodenum showed no sign of gallstones up.

**Fig. 2:**
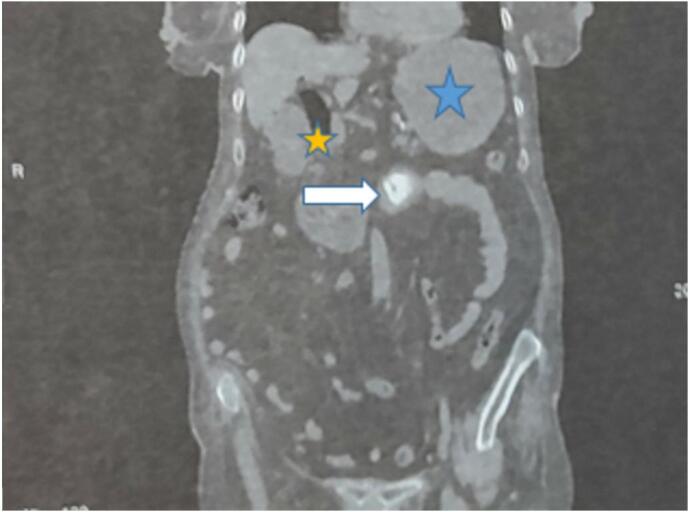
CT scan showing Rigler triad: pneumobilia/ gastric distention /large gallstone in dudenojujenum junction.

An 82-year-old female patient with a history of coronary artery disease and coronary stenting, known to have gallstones, presented with a nausea vomiting and post prandial abdominal pain evolving for two weeks. Additionally, the patient presented with a pulse rate of 102 beats per minute, a weak pulse, blood pressure measuring 10/6, and indicators of extracellular dehydration, such as a persistent mucosal fold, clammy skin with decreased elasticity and sunken eyes. Laboratory tests revealed the presence of hypokalemia and hypochloremia. After putting the urinary catheter we noticed a reduced urinary output. We initiated resuscitation by inserting a nasogastric tube, which promptly drained 400 cc of gastric fluid. The fluid resuscitation was performed based on the input-output assessment, aiming to correct any hydro electrolytic imbalances. After stabilizing the patient an esophagogastroduodenoscopy revealed normal findings up to the second part of the duodenum [Fig. 1], there was no duodenal dilatation and the EGD showed some retained bile. Regarding the symptomatology and signs of gastric outlet obstruction searching for ethiology we decided to perform a complementary abdominal computed tomography that showed a cholecystoduodenal fistula with a measured 4 cm stone lodged at the duodenojejunal jonction [Fig. 2]. Due to the absence of endoscopic treatment at that time, the decision was made to proceed with surgery.

**Fig. 3:**
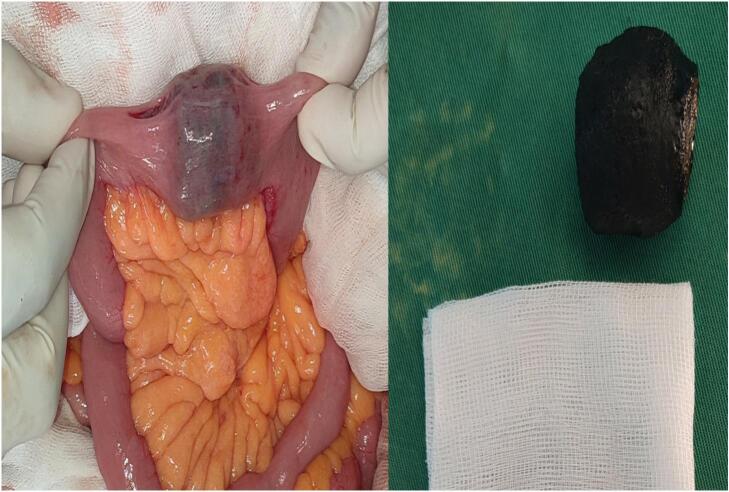
The stone successfully advanced to the second jejunal loop and removed.

During laparotomy, a lodged stone was found at the duodenojejunal angle. The stone was successfully advanced to the second jejunal loop [Fig. 3], where an enterotomy was performed, allowing for the extraction of the stone. After that we decided using this enterotomy, on the second jejunal loop, to proceed with a latero-lateral gastroenteroanastomosis Trans- and sub-mesocolic, without gastric resection. The cholecystoduodenal fistula was left untouched.

Postoperative recovery was uneventful, and the patient was discharged on the 8th day post-surgery. 3 month follow up was uneventful.

## 3 Discussion

Gallstone ileus is an uncommon condition, accounting for 1 % of all causes of small-bowel obstruction, but it may be the diagnosis in up to 25 % of patients older than 65 years who present with small-bowel obstruction in the absence of a hernia [3].

Bouveret Syndrome is characterized by the impaction of a gallstone, which has either passed into the duodenum or pyloric channel via a cholecystoduodenal or cholecystogastric fistula. The risk factors for Bouveret syndrome includes old age (>70 years), female gender, gallstones >2.5 cm, and post-surgical altered gastrointestinal anatomy [1,3]. Our patient was an 82 years old female with a coronary stenting and a history of untreated gallstones.

The challenge in diagnosing Bouveret Syndrome lies in its rarity and the nonspecific nature of its symptoms, which can be easily mistaken for other gastrointestinal disorders. Therefore it is crucial to be considered and to be differentially diagnosed from other causes of gastric outlet obstruction, such as gastric cancer and peptic stenosis especially in elderly women [4].

Severe upper abdominal pain, accompanied by persistent nausea and vomiting, are a hallmark of the condition other clinical manifestation such as recent weight loss, anorexia [5] and constipation are reported. Signs of dehydration and abdominal distension may be observed, reflecting the obstructed flow within the gastrointestinal tract. Our patient reported vomiting of a gastric stasis fluid, anorexia and bloating suggesting gastric outlet obstruction.

The esophagogastroduodenoscopy (EGD) is a key exam to eliminate differential diagnosis and to confirm the presence of gallstones in stomach or in the duodenum but It is important to keep in mind that in patients diagnosed with Bouveret syndrome, only approximately two-thirds of stones can be visualized on diagnostic EGD [4,5]. The EGD for our patient showed no sign of gallstones up to the second part of the duodenum.

Radiological imaging plays a pivotal role in confirming Bouveret Syndrome. Abdominal plain radiography can demonstrate in 10–50 % of the cases demonstrates the elements of the Rigler triad: bowel obstruction, pneumobilia and a calcified ectopic gallstone [1,4]. While abdominal ultrasound aids in identifying gallstones. We didn't request an abdominal plain radiography nor an abdominal ultrasound for our patient; she was immediately examined through a Computed tomography (CT) scan.

CT scan is the best imaging exam that can provide detailed images, it helps visualizing the impacted gallstone and its location within the digestive system. It can show the Rigler triad in 75 % of cases [6].The abdominal tomography showed a cholecystoduodenal fistula with a measured 4 cm stone lodged at the duodenojejunal angle, pneumobilia and stomach obstruction. The localization of the stone explains the absence of gallstones in the EGD.

The management of Bouveret Syndrome often requires a multidisciplinary approach. Initial stabilization involves supportive care, with intravenous fluids and pain management to relieve symptoms. The primary goal is to alleviate the obstruction by removing the impacted stone. This can be achieved endoscopically, surgically or by using other techniques such as endoscopic electrohydraulic lithotripsy, and extracorporeal shockwave lithotripsy [7]. Modern management focuses on less invasive techniques taking into account the advanced age and serious concomitant illnesses of the majority of the affected patients. When endoscopic treatment is not available such in our case surgical intervention is a common strategy, with open surgery or laparoscopic procedures employed to remove the obstructing gallstone.

The optimal surgical management of gallstone ileus is related to the management of the cholecystoduodenal fistula and it remains a subject of debate within the medical community. To address these concerns, two alternative surgical approaches have been proposed. The first is a one-stage procedure involving enterolithotomy, cholecystectomy, and simultaneous fistula repair during the same operation [8,9]. The second is a two-stage procedure, comprising enterolithotomy initially, followed by interval cholecystectomy with fistula repair once the patient has recovered from the acute episode [10]. In order to answer this question a study conducted by Reisner and Cohen compared the mortality rates between patients undergoing enterolithotomy alone and those undergoing the one-stage procedure, which includes enterolithotomy, cholecystectomy, and fistula repair concurrently. The findings revealed a mortality rate of 11.7 % in patients subjected to enterolithotomy alone, whereas those undergoing the one-stage surgery exhibited a mortality rate of 16.9 %. Despite a recurrence rate of approximately 5 % in gallstone ileus cases, enterolithotomy alone may be the preferred treatment, especially for patients with hemodynamic instability or significant comorbidities [11]. Others studies fuel the debate by exposing that fistula repair may be unnecessary because the fistula could spontaneously close especially if the cystic duct is patent and residual gallstones are not present [3,5].

In our case we started moving the stone by milking it to the jejunum then we performed an enterotomy through the second jejunal loop to extract the gallstone witch was 4 cm in size. We preferred alatero-lateral gastroenteroanastomosis Trans- and sub-mesocolic without gastric resection after the gallstone extraction. Nevertheless it can be controversial but we thought that performing this latero-lateral anastomosis may reduce the risk of recurrence of gastric outlet obstruction, with neither touching the cholecystoduodenal fistula and nor exposing the patient to significant rate of biliary fistula especially considering the patient's condition and comorbidities. Although this alternative may not prevent the patient from potentially recurrent gallstone ileus.

During the operation the whole intestine should be examined, since in 16 % of the cases other gallstones are present at another location in the digestive tract [1].

Bouveret Syndrome poses unique challenges due to its rarity, often leading to delayed diagnosis and treatment initiation. The complexity of the condition, coupled with the need for a tailored approach, necessitates collaboration among various medical specialties, including gastroenterology and surgery. The prognosis for patients with Bouveret Syndrome varies, depending on factors such as the size and location of the impacted stone, the patient's overall condition, and the chosen treatment modality. Early intervention and a comprehensive care plan are critical for achieving favorable outcomes. At 3 month follow up the patient was seen and was satisfied with the procedure.

## 4 Conclusion

Bouveret Syndrome, though rare, demands attention and awareness. Timely diagnosis and appropriate management are essential to improve patient outcomes. EGD and mostly CT scan provides the diagnosis. Endoscopic removal of gallstone may be proposed as a first line approach if not available surgery remains the only treatment. Enterotomy or gastrotomy can be proposed. The treatment of the fistula remains the greater controversial issue; a one stage or a two stage procedure are options depending on the patient stability and comorbidities.

## Ethical approval

Our institutions “la Rabta Hospital” and “School of Medicine of Tunis” require no ethical approval for case reports. It is required for studies on human participants. This is just a case report with written patient approval.

## Funding

No funding.

## Author contribution

Souhaib Atri: conceptualization, data curation, redaction, project manager.

Elaifia Rany: conceptualization, data curation, redaction, project manager.

Amine Sebai: conceptualization, redaction.

Hammami.M: resources, visualization.

Haddad.A: resources, visualization.

Montassar Kacem: supervision, validation, visualization.

## Guarantor

Elaifia rany.

## Research registration number

Not applicable.

## Conflict of interest statement

All authors declare that they have no conflicts of interest.
